# Salicylic Acid as a Febrifuge

**Published:** 1876-08

**Authors:** 


Salicylic Acid as a Febrifuge.—Prof. C. Binz states (*The Practitioner*, June, 1876,) that "Salicylic acid possesses in all outward respects a resemblance to quinine. It combats the malarial poisoning (Senator, Buss)—although less surely and effectually than quinine—during the period of apyrexia, where, as is well known, neither the pulse nor respiration necessarily present the slightest abnormity. It is, like quinine, a powerful antizymotic, which can be introduced into the organism in large quantities, circulates there a considerable length of time, and is given off again—at least partially—in an unchanged condition. Even the ringing in the ears and the slight deafness characteristic of cinchonism, are not wanting in connection with the medication with salicylic acid. A complete agreement between its behavior and that of quinine toward certain disease-producing agencies, known, as must be confessed, only by their effects, does not exist. This we have already seen in speaking of intermittent fever. Acute articular rheumatism, a disease in which quinine avails so little and salicylic acid so much, furnishes a second and converse example. Considering the chemical dissimilarity of the two substances, it is not to be expected that there should exist a similarity between their modes of action on more than the general points involved. This general resemblance, however, is unquestionably present, and so we shall have to seek an explanation of the manner in which their therapeutic action is exerted in the same channels."—*Am. Jour. Med. Sciences.*

